# Intrapancreatic accessory spleen

**DOI:** 10.1590/S1679-45082017RC3942

**Published:** 2017

**Authors:** Marcelo Protásio dos Santos, Aline Pacheco de Rezende, Paulo Vicente dos Santos, José Eduardo Gonçalves, Fernando Bray Beraldo, Adriano Pereira Sampaio

**Affiliations:** 1 Hospital do Servidor Público Estadual “Francisco Morato de Oliveira”, São Paulo, SP, Brazil.; 2 Hospital Universitário, Universidade Federal de Sergipe, Aracaju, SE, Brazil.

**Keywords:** Spleen, Pancreas, Neuroendocrine tumors/diagnosis, Diagnosis, differential, Case reports, Baço, Pâncreas, Tumores neuroendócrinos/diagnóstico, Diagnóstico diferencial, Relatos de casos

## Abstract

An asymptomatic 79-year-old woman, with incidental finding on abdominal ultrasound of a solid nodule in the tail of the pancreas. Magnetic resonance imaging showed a 12mm solid tumor. The suggested diagnosis was pancreatic neuroendocrine tumor. The pathological examination showed an intrapancreatic splenic tissue. This is a rare ectopic location of spleen tissue and it should be considered in the differential diagnosis of pancreatic solid tumors.

## RESUMO

Mulher de 79 anos, com achado incidental em ultrassonografia de abdome de lesão sólida em cauda de pâncreas. Em ressonância magnética, foi confirmada lesão de 12mm. Foi aventada a hipótese diagnóstica de tumor neuroendócrino não secretor, sendo realizada pancreatectomia distal laparoscópica. Em estudo anatomopatológico, diagnosticou-se tecido esplênico, configurando baço acessório intrapancreático. Este tipo de manifestação topográfica de baço é rara, mas deve fazer parte dos diagnósticos diferenciais em tumores sólidos do pâncreas.

## INTRODUCTION

The accessory spleen is a congenital anomaly caused by a failure in embryologic development of spleen tissue with occurring in 10% of general population. In 16% of cases, this tissue is found in tail of the pancreas.^(1)^

Intrapancreatic accessory spleen is a benign affection and rarely symptomatic. This affection great importance relays on the fact that of being a differential diagnosis to pancreatic neuroendocrine, but with therapies and completely different prognosis.^(2)^

We report a case of an incidental finding in radiology exam that mimicked a neuroendocrine pancreatic tumor.

## CASE REPORT

This was an asymptomatic 79-year-old woman followed-up for chronic C hepatitis and who after an abdominal ultrasound had an incidental finding of a hypoechoic nodule measuring 10mm in the tail of the pancreas. Her physical exam did not show changes and laboratorial tests, including the cancer antigen 19.9, were normal.

**Figure 1:**
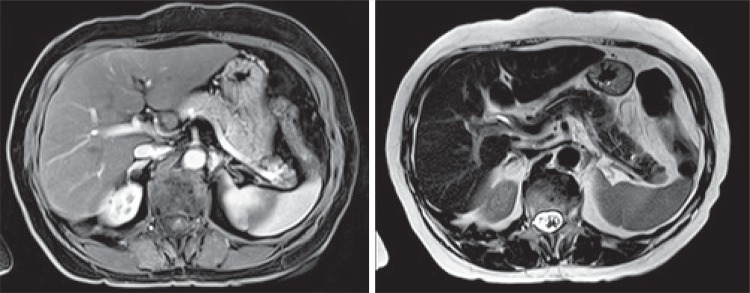
Abdominal magnetic resonance imaging (T1 and T2)

Abdominal magnetic resonance confirmed the finding of nodular injury measuring 12mm in the tail of the pancreas, hypointense in T1, hyperintense in T2, and highlight after infusion of contrast agent (Figure 1).

**Figure 2:**
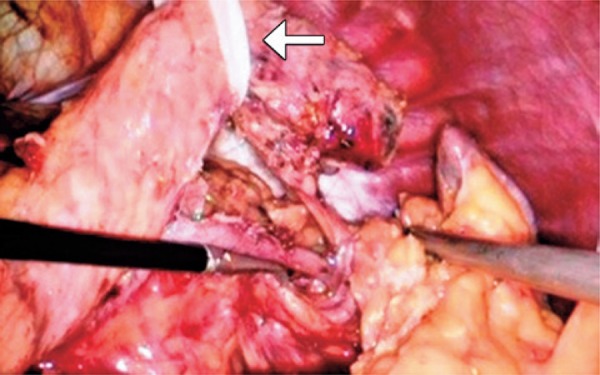
Intraoperative with repaired pancreas and isolated splenic vessels

Based on these findings, we suspected of non-functioning neuroendocrine pancreatic tumor. A distal pancreatectomy was carried out with spleen preservation (Figure 2). The surgical procedure was done without intercurrences by videolaparoscopic access, and pancreatic resection with yellow reload linear stampler (EndoGIA^®^) with 60mm. The patient evolved with pancreatic fistula in the fifth day after surgery without need of surgery. She was discharged 16 days after the surgery.

**Figure 3:**
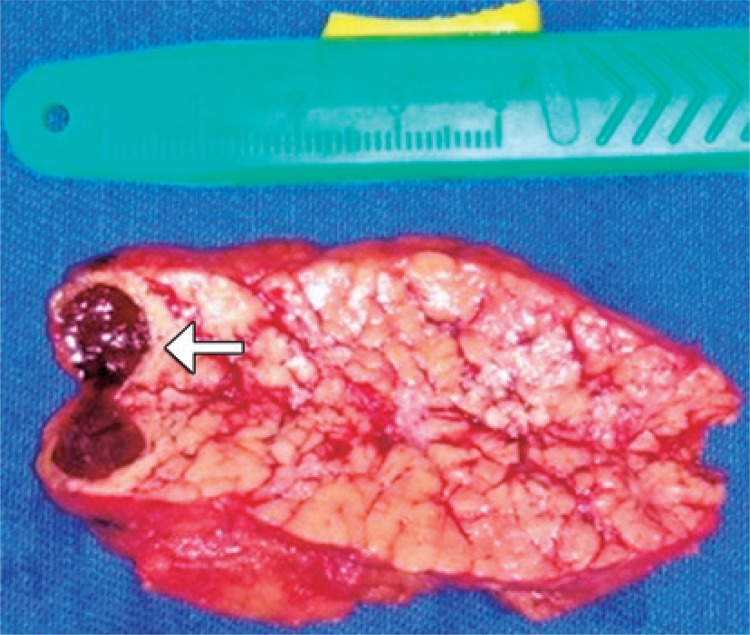
Surgical sample of distal pancreatectomy with intrapancreatic ectopic spleen

Mascroscopic cuts of surgical specimen showed nodular area of smooth aspect and brown color with 12x7x7mm surrounded by pancreatic tissue. The pathological examination confirmed the diagnosis of accessory intrapancreatic spleen (Figure 3).

## DISCUSSION

Accessory spleen is a congenital abnormality with incidence approximately 10% in necropsy studies.^(1)^ Its development results in a change during differentiation of mesenchymal cells in formation of splenic tissue through the trajectory of splenic vessels.^(2)^ Normally ectopic spleens (around 80%) are placed closed to splenic hilum, and 16% are located in the tail of the pancreas.^(3)^

In general, intrapancreatic accessory spleen is an asymptomatic injury and without need of surgical therapy. However, in the majority of described cases, diagnosis was made after surgical resection because of the difficult in the pre-operative differential diagnosis with pancreatic neuroendocrine tumors.^(4)^

Pancreatic neuroendocrine tumors are rare neuroendocrine neoplasias with an annual incidence estimation of <1/100,000 in population studies and correspond for less than 2% of all pancreatic tumors.^(5,6)^ These tumors are classified as functioning and non-functioning according to hormonal secretion and symptoms observed, but, unfortunately, there are no global consensus to such definition. Most of pancreatic neuroendocrine tumors is no-functioning and the majority of them are malignant.^(6)^Primary surgical resection is an associated factor with increase in long-term survival in these tumors, mainly in injuries greater than 20mm. The conservative therapy is preferred in cases of tumors smaller than 10mm.^(7)^

The significant importance to differentiate intrapancreatic accessory spleen from pancreatic neuroendocrine tumors before the surgery relays on the fact that the latter needs surgical intervention whereas the first should be approached conservatively. The difficulty is to perform differential diagnosis, mainly because, so far, there are no laboratorial or radiologic exams to confirm or exclude the diagnosis of intrapancreatic accessory spleen.^(4)^

Imaging exams can be useful to differentiate two injuries. However, computed tomography with contrast agent and conventional magnetic resonance are limited in this evaluation mainly in injuries smaller than 10mm.^(8)^

Magnetic resonance combined with diffusion-weighted phase revealed to be a high accurate method in diagnosis and differentiation of intrapancreatic ectopic spleen and small solid pancreatic tumors. The intrapancreatic ectopic spleen normally is seen in magnetic resonance of hyperintense diffusion-weighted in T2 and hypointense weighted in T1 compared to normal pancreatic tissue.^(8)^ The endoscopic ultrasound with aspiration biopsy, in addition to images, provides a definitive diagnosis by the use of pathological examination, but it constitutes an exam that depends on the researcher and injury site, in addition to be an invasive method.^(9)^

The ^68^Ga-DOTA-TOC PET/CT is a high specificity method for diagnosis of pancreatic neuroendocrine tumors because there is an important expression of receptors of somatostatin on lymphocytes. Therefore, a physiological accumulation of ^68^Ga-DOTA-TOC is always seen in splenic tissue.^(10)^Scintigraphy with marked erythrocytes with 99 technetium is one of the most specific methods to diagnose intrapancreatic ectopic spleen, because when marked erythrocytes are injected with radiopharmacy, more than 90% of the material is uptake by the splenic tissue, therefore contributing significantly to detect intrapancreatic splenic tissue and differentiate neuroendocrine pancreatic tumors and, mainly, avoid unnecessary surgical procedure.^(11)^

## CONCLUSION

Intrapancreatic ectopic spleen is rare. Surgery is not indicated in asymptomatic patients. This affection must be considered as a differential diagnosis before surgery of solid pancreatic injuries suggestive of neuroendocrine neoplasias to avoid unnecessary pancreatic resections.
